# Hollow Hemispherical Lithium Iron Silicate Synthesized by an Ascorbic Acid-Assisted Hydrothermal Method as a Cathode Material for Li Ion Batteries

**DOI:** 10.3390/ma15103545

**Published:** 2022-05-16

**Authors:** Huaifu Li, Yunsong Li, Xuan Cheng, Chaoyang Gong

**Affiliations:** 1 Department of Materials Science & Engineering, College of Materials, Xiamen University, Xiamen 361005, China; 20720171150018@stu.xmu.edu.cn (H.L.); gcy@xmu.edu.cn (C.G.); 2 School of Materials Science and Engineering, Northwestern Polytechnical University, Xi’an 710072, China; 3 Fujian Key Laboratory of Advanced Materials, Xiamen University, Xiamen 361005, China

**Keywords:** Li ion batteries, cathode material, hollow hemispherical Li_2_FeSiO_4_, ascorbic acid

## Abstract

High-capacity and high-voltage cathode materials are required to meet the increasing demand for energy density in Li ion batteries. Lithium iron silicate (Li_2_FeSiO_4_) is a cathode material with a high theoretical capacity of 331 mAh·g^−1^. However, its poor conductivity and low Li ion diffusion coefficient result in poor capability, hindering practical applications. Morphology has an important influence on the properties of materials, and nanomaterials with hollow structures are widely used in electrochemical devices. Herein, we report a novel hollow hemispherical Li_2_FeSiO_4_ synthesized by a template-free hydrothermal method with the addition of ascorbic acid. The hollow hemispherical Li_2_FeSiO_4_ consisted of finer particles with a shell thickness of about 80 nm. After carbon coating, the composite was applied as the cathode in Li ion batteries. As a result, the hollow hemispherical Li_2_FeSiO_4_/C exhibited a discharge capacity as high as 192 mAh·g^−1^ at 0.2 C, and the average capacities were 134.5, 115.5 and 93.4 mAh·g^−1^ at 0.5, 1 and 2 C, respectively. In addition, the capacity increased in the first few cycles and then decayed with further cycling, showing a warm-up like behavior, and after 160 cycles the capacities maintained 114.2, 101.6 and 79.3 mAh·g^−1^ at 0.5, 1 and 2 C, respectively. Such a method of adding ascorbic acid in the hydrothermal reaction can effectively synthesize hollow hemispherical Li_2_FeSiO_4_ with the enhanced electrochemical performance.

## 1. Introduction

Rechargeable batteries with a high energy density, high power density, good rate performance, long cycle life and a reasonable price are an important part of sustainable energy, and their gradual application in energy-consuming devices will significantly alleviate the environmental problems caused by fuel systems [1]. Li ion batteries are mature commercial rechargeable batteries, but their energy density, cycle life and safety performance need to be improved to meet the needs of higher energy density, longer battery life and safer performance. Cathode materials dominate the energy density of Li ion batteries, and commercial cathode materials LiCoO_2_ and LiFePO_4_ deliver capacities below 170 mAh·g^−1^. Therefore, it is necessary to develop cathode materials with the potential to release higher capacities. Li_2_FeSiO_4_ has the advantage of a high theoretical capacity (331 mAh·g^−1^), environmental friendliness and a relatively low cost [2], and its cycle performance is better than that of Li_2_MnSiO_4_ [3] since Li_2_MnSiO_4_ suffers from the crystal structural deformation caused by the Jahn-Teller effect of Mn^3+^ [4,5]. However, the low ionic diffusion coefficient (~16 × 10^−16^ cm^2^/s) [6] and poor electronic conductivity (~6 × 10^−14^ S·cm^−1^) [7] make it difficult for Li_2_FeSiO_4_ to deintercalate more than one Li ion (corresponding to 165 mAh·g^−1^) at room temperature. In addition, the deintercalation of the second Li ion in Li_2_FeSiO_4_ originates from the oxidation of O^2−^ rather than the oxidation of Fe^3+^ [2,8,9], since the oxidation potential of Fe^3+^/Fe^4+^ is higher than 4.8 V, O^2−^ with a lower oxidation potential oxidized first [2,10,11]. To achieve a high capacity, most of the modified Li_2_FeSiO_4_ materials have been prepared by the strategies of ion doping [12,13,14], morphology control [15,16,17], preparation of nanocrystals [18,19,20] or the methods combined with carbon coating [21,22,23,24,25,26].

Material morphology has an important influence on the performance, and a high porosity morphology endows the material with efficient electrolyte penetration and fast electron transport [27]. Hydrothermal synthesis enables the fabrication of Li_2_FeSiO_4_ nanostructures with well-defined shapes and fine-tuned dimensions [27]. Hollow spherical structures have the advantage of a large specific surface area, large voids, and strong mass transfer ability, which can improve the performance of low-kinetic, high-capacity cathode materials [28,29]. However, the hydrothermally synthesized Li_2_FeSiO_4_/C composites with hollow spherical structures exhibited capacities lower than 180 mAh·g^−1^ at 0.1 C and 165 mAh·g^−1^ at 0.2 C [16,24,30,31,32,33]. The discharge capacity of hollow spherical Li_2_FeSiO_4_ synthesized by a hydrothermal method was 120 mAh·g^−1^ at 0.1 C and less than 40 mAh·g^−1^ at 2 C [16], and that of hollow mesocrystal Li_2_FeSiO_4_ decayed from 162 to 150 mAh·g^−1^ after six cycles (55 °C, 1/50 C) [34].

In this work, a novel hollow hemispherical Li_2_FeSiO_4_ was synthesized by a hydrothermal method with the addition of ascorbic acid. It has been found that ascorbic acid inhibited the oxidation of Fe^2+^ and reduced the particle size [35], and acted as a reducing agent [34,36]. Different to the previous studies, we demonstrate that the ascorbic acid significantly alters the particle morphology, enabling primary particles to self-assemble into hollow hemispherical structures with the enhanced electrochemical performance.

## 2. Materials and Methods

### 2.1. Materials Synthesis

The materials were synthesized by a hydrothermal method. A typical synthesis process is as follows. 0.02 mol FeCl_2_∙4H_2_O (99.7%, Tianjin Guangfu Fine Chemical Research Institute, Tianjin, China) was dissolved in a mixed solution of 10 mL ethylene glycol and 50 mL deionized water to form a light green solution, and then 0.02 mol hydrophilic SiO_2_ (99.8%, Shanghai Macklin Biochemical Co., Ltd., Shanghai, China) was slowly added and stirred evenly. Then, 0.0031 mol ascorbic acid (C_6_H_8_O_6_, 99.7%, Xilong Scientific Co., Ltd., Shantou, China) was added, and finally, 0.12 mol LiOH∙H_2_O (Sinopharm Chemical Reagent Co., Ltd., Shanghai, China) was added, and stirred to form a dark green suspension. The suspension was transferred to a reaction kettle, heated from room temperature to 160, or 180 or 200 °C, and maintained for 12 h to obtain a grey sediment. The sediment was washed with alcohol and deionized water, and was centrifuged to obtain a yellow solid, which was then vacuum-dried at 80 °C for 6 h. Three samples obtained after hydrothermal reactions at 160, 180 and 200 °C are denoted as LFS160, LFS180 and LFS200, respectively. A sample obtained after a hydrothermal reaction without the addition of ascorbic acid at 200 °C is denoted as nano-LFS200. The LFS200 (or nano-LFS200) was mixed with sucrose (with a mass ratio of 1:2) in a beaker containing 10 mL deionized water and 10 mL ethanol, and the mixture was magnetically stirred at 80 °C. After solvent evaporation, a solid was collected and then calcined at 600 °C for 10 h in a nitrogen atmosphere to obtain LFS600 (or nano-LFS600).

### 2.2. Materials Characterization

Powder X-ray diffraction (XRD) patterns in the range of 2*θ* = 10–80° (10°⋅min^−1^) were recorded on an X-ray diffractometer (Ultima-IV, Rigaku, Tokyo, Japan) using Cu Kα radiation (λ = 0.154 nm) to study the phase composition and crystal structure of the materials. Morphological and microstructural observations were conducted by a field emission scanning electron microscope (FE-SEM, S-4800, Hitachi, Tokyo, JapanJapan), and a transmission electron microscope (TEM, Talos F200S, FEI, Eindhoven, Netherlands) equipped with an energy-dispersive X-ray spectrometer (EDX, Super-X, FEI, Eindhoven, Netherlands). Selected area electron diffraction (SAED) and high-resolution transmission electron microscopic (HRTEM) and EDX mapping measurements were performed on the transmission electron microscope operated at 200 kV.

### 2.3. Electrodes Fabrication and Electrochemical Tests

Electrodes were fabricated by scribbling a mixture of the as-prepared LFS600 (or nano-LFS600), super-P and PVDF (a mass ratio of 8:1:1) on an Al foil, and an appropriate amount of N-methyl-2-pyrrolidone (NMP) was used as a solvent for the mixture. The electrodes were dried in a vacuum oven at 60 °C for 12 h. Cyclic charge–discharge measurements were performed on 2016-type coin cells at 30 °C in a potential window of 1.5–4.8 V versus Li/Li^+^ using a Land BT-2000 test system (LAND Electronic Co., Ltd., Wuhan, China). The coin cells were assembled in an Ar-filled glovebox (Mbraun, Garching, Germany). An electrode was used as the cathode, a sheet of lithium metal was used as the anode, and a polypropylene membrane (Celgard 2500) was used as the separator. The electrolyte in the cells was composed of 1 M LiPF_6_ dissolved in ethylene carbonate (EC), ethyl methyl carbonate (EMC) and dimethyl carbonate (DMC) (a volume ratio of 1:1:1) with 2% fluoroethylene carbonate (FEC). Electrochemical impedance spectroscopic (EIS) data were collected with an Autolab PGSTAT101 (Metrohm, Barendrecht, Netherlands) in the frequency range from 100 kHz to 0.1 Hz at an amplitude of 10 mV.

## 3. Results and Discussion

### 3.1. Structural Analysis

**Figure 1:**
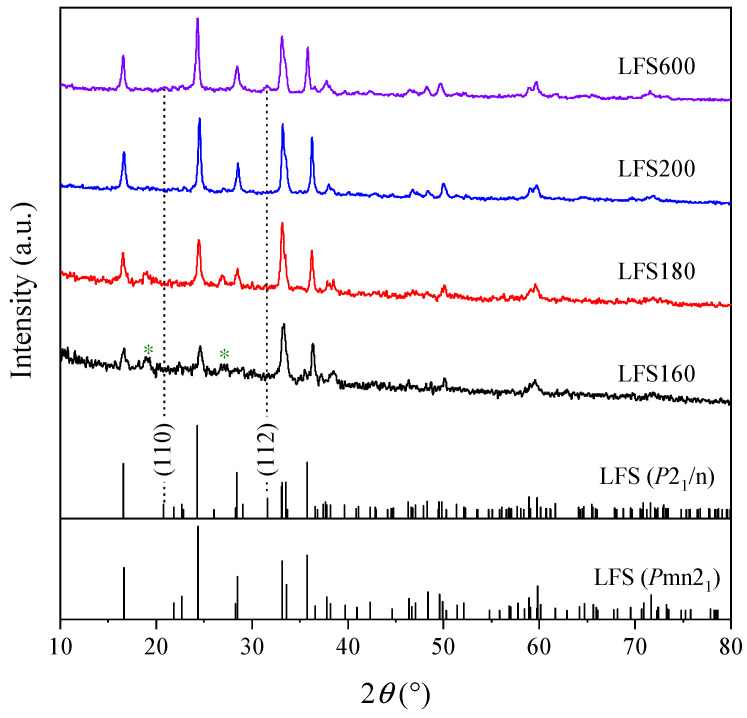
XRD patterns of the as-prepared Li_2_FeSiO_4_ samples. The diffraction peaks marked with an asterisk “*” represent the impure phase of Li_2_SiO_3_. The standard lines of monoclinic Li_2_FeSiO_4_ (*P*2_1_/n space group, PDF#97-024-6132) and orthorhombic Li_2_FeSiO_4_ (*P*mn2_1_ space group, PDF#97-016-1306) are included for comparison.

XRD patterns of the as-prepared samples are presented in Figure 1. The standard lines of monoclinic Li_2_FeSiO_4_ (*P*2_1_/n space group, PDF#97-024-6132) and orthorhombic Li_2_FeSiO_4_ (*P*mn2_1_ space group, PDF#97-016-1306) are also included for comparison. The results show that the orthorhombic Li_2_FeSiO_4_ structure was formed through hydrothermal reactions at 160, 180 and 200 °C, which is consistent with that of the Li_2_FeSiO_4_ obtained by the hydrothermal reaction at 200 °C without adding ascorbic acid [37]. The characteristic diffraction peaks observed from LFS600 in Figure 1 were ascribed to the (110) and (112) crystal planes of monoclinic Li_2_FeSiO_4_ (*P*2_1_/n space group), indicating a phase evolution from orthorhombic to monoclinic [37].

**Figure 2:**
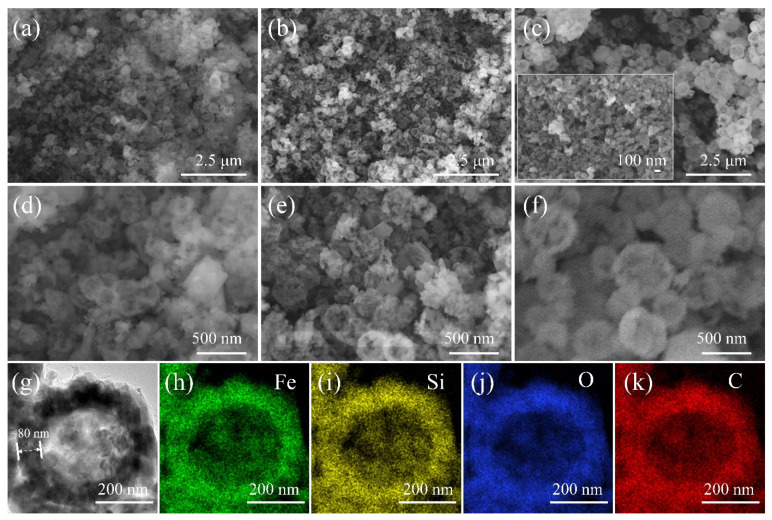
Morphologies of the Li_2_FeSiO_4_ samples. (**a**–**f**) SEM images of LFS160 (**a**,**d**), LFS180 (**b**,**e**) and LFS200 (**c**,**f**) (the inset in (**c**) is nano-LFS200). (**g**) TEM image and (**h**–**k**) EDX mapping images of Fe, Si, O and C for LFS600.

**Figure 3:**
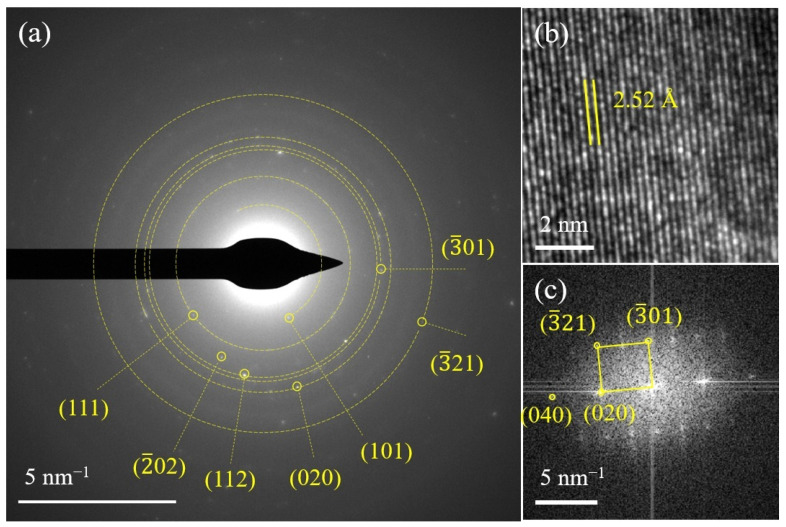
Microstructure characterization of LFS600. (**a**) SAED, (**b**) HRTEM and (**c**) FFT images.

SEM images given in Figure 2a–f demonstrate that the LFS160, LFS180 and LFS200 exhibited a hollow hemispherical morphology. Figure 2e indicates that hollow hemispheres were composed of smaller nanoparticles. Particle size distribution diagrams shown in Figure S1 revealed the average sizes of 200–250, 250–320 and 450–550 nm for the LFS160, LFS180 and LFS200, respectively, were based on 100 particles on each sample. The higher the temperature, the larger the particle size. The inset in Figure 2c presents the morphology of sample nano-LFS200, obtained by the hydrothermal synthesis without ascorbic acid, and the relatively fine nanoparticles were observed. The results show that ascorbic acid changed the morphology, promoting self-assembly of primary particles into secondary hollow hemispherical particles. Ascorbic acid was used as a reducing agent in the hydrothermal synthesis of Li_2_FeSiO_4_ [34,36], and could reduce the particle sizes of Li_2_FeSiO_4_ [35] and Ag [38]. Moreover, ascorbic acid could regulate the particle self-assembly behavior of Li_2_FeSiO_4_. Figure 2g shows that the LFS600 exhibited a hollow hemispherical structure with a shell thickness of about 80 nm. EDX mapping images (Figure 2h–k) reveal that the elements of Fe, Si and O were uniformly distributed on the hollow hemispherical particle that was uniformly coated by C. A SAED image (Figure 3a) shows that the diffraction spots with distances of 5.37, 3.65, 3.13, 2.81, 2.68, 2.48 and 1.87 Å from the diffraction center are ascribed to the (101), (111), ($\bar{2}$02), (112), ($\bar{3}$01), (020) and ($\bar{3}$21) crystal planes, respectively. The lattice fringes with a lattice spacing of 2.52 Å (Figure 3b) are assigned to the (020) plane. The diffraction spots in the FFT image (Figure 2c) are ascribed to the ($\bar{3}$01), (020) and ($\bar{3}$21) planes of monoclinic Li_2_FeSiO_4_. The observed (112) crystal plane of monoclinic Li_2_FeSiO_4_ indicates that the obtained LFS600 is predominated by monoclinic Li_2_FeSiO_4_, which confirms the results of XRD.

### 3.2. Electrochemical Performance

Considering that the samples LFS160 and LFS180 contained an impure phase of Li_2_SiO_3_ (Figure 1), and their particle development degree (Figure 2d,e) was lower than that of LFS200 (Figure 2f), the follow-up study was only focused on the LFS200. The hydrothermally synthesized Li_2_FeSiO_4_ precursors commonly exhibit poor conductivity, and the carbon-coating and calcination are necessary for the electrochemical performance measurements [16,39,40,41]. Thus, the LFS200 sample was further mixed with sucrose and calcined at 600 °C to improve the conductivity (denoted as LFS600).

**Figure 4:**
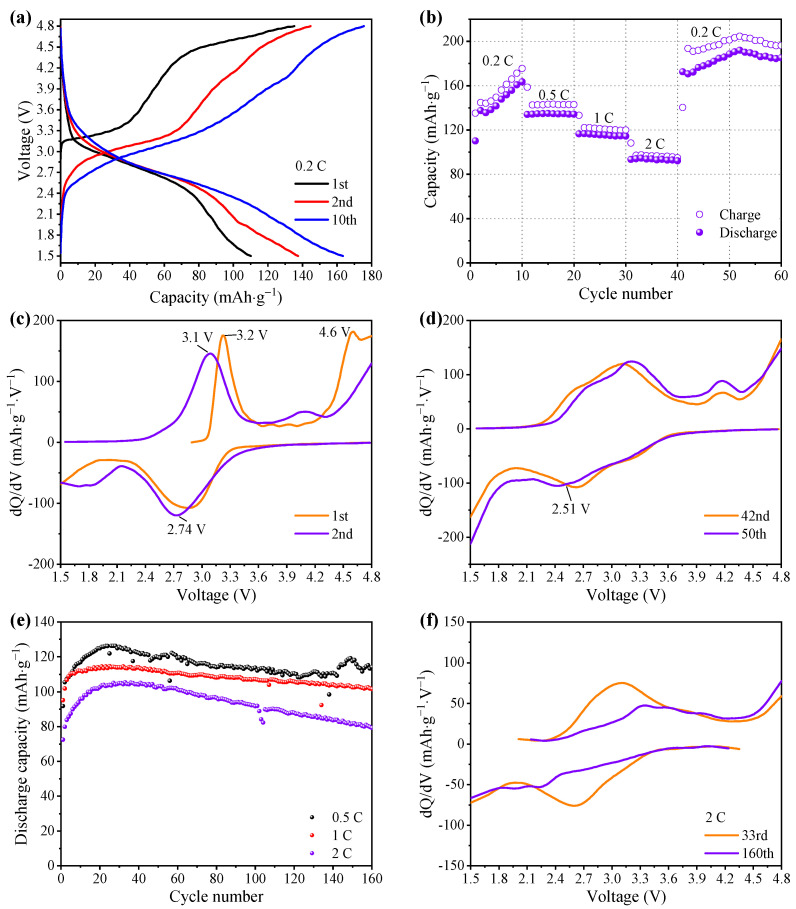
Electrochemical performance of the sample LFS600 at 1.5–4.8 V and 30 °C (**a**) Charge–discharge curves of the 1st, 2nd and 10th cycles at 0.2 C, (**b**) rate performance (1 C = 166 mA·g^−1^), differential capacity curves of (**c**) the 1st and 2nd cycles, and (**d**) the 42nd and 50th cycles, (**e**) cycle performance at 0.5, 1 and 2 C, and (**f**) differential capacity curves for the 33rd and 160th cycles.

The charge–discharge curves of LFS600 are shown in Figure 4a. The first charge curve showed a voltage plateau at 3.2 V, attributed to Fe^2+^/Fe^3+^ oxidation [42]. When the voltage exceeded 4.2 V, another voltage plateau was formed due to the O^2−^ oxidation [43], which is similar to those caused by the oxidation of O^2−^ on Li-rich cathode materials [44,45] and Li_1.25_Nb_0.25_Fe_0.50_O_2_/C [46]. The first discharge curve became smooth at ~3.3 V, and then a plateau was formed, which is caused by the Fe^3+^/Fe^2+^ reduction. The discharge plateau corresponding to the reduction peak at ~2.74 V in the differential capacity curve is presented in Figure 4c. The 2nd and 10th charging curves show a decreased curvilinear polarization, ostensibly rising from a capacity increase. Compared with the hollow spherical Li_2_FeSiO_4_ reported previously [16], the hollow hemispherical Li_2_FeSiO_4_ prepared with the addition of ascorbic acid exhibited a smaller curvilinear polarization.

Rate performance of the LFS600 (Figure 4b) show that the discharge capacity at 0.2 C increased from 110.1 mAh·g^−1^ (1st) to 163.4 mAh·g^−1^ (10th), which is ostensibly caused by the decreased curvilinear polarization, which is essentially attributed to the material activation [47]. The average discharge capacities at 0.5, 1 and 2 C were 134.5, 115.5 and 93.4 mAh·g^−1^, respectively, while those of the nano-LFS600 (without ascorbic acid) were 121.9, 110.5 and 96.9 mAh·g^−1^, respectively (Figure S2). Both the LFS600 and nano-LFS600 delivered a better performance than those of the nanoparticles sized 50–100 nm (135, 117 and 87 mAh·g^−1^ at 0.2, 0.5 and 1 C, respectively) [48]. When the rate changed from 2 C to 0.2 C, the discharge capacity increased from 172.5 mAh·g^−1^ (41st) to 185.8 mAh·g^−1^ (50th), which is higher than that of the hollow spherical Li_2_FeSiO_4_ (its maximum capacity was 168.1 mAh·g^−1^ at 0.1 C, and below 150 mAh·g^−1^ at 0.2 C) [30]. Additionally, a hollow spherical Li_2_FeSiO_4_ prepared by a template-free method, delivered the capacity less than 120 mAh·g^−1^ (0.1 C, 1.5–4.8 V) [16], a Li_2_FeSiO_4_ prepared with a template 168.1 mAh·g^−1^ (0.1 C, 1.5–4.6 V) [30], and a spherical Li_2_FeSiO_4_ with a template 140 mAh·g^−1^ at 0.2 C [31]. As shown in Table S1, the hollow hemispherical Li_2_FeSiO_4_ prepared with an ascorbic acid-assisted template-free method had a good electrochemical performance.

To analyze the reason for capacity increase from the 42nd cycle to the 50th cycle, differential capacity curves are compared in Figure 4d. The reduction curve of the 50th cycle has a greater |dQ/dV| value at 2.51–1.5 V than that of the 42nd cycle, indicating a faster reduction reaction at 2.51–1.5 V. This result reflects that the increase in capacity from the 42nd cycle to the 50th cycle is attributed to the accelerated reduction reaction at 2.51–1.5 V, caused by a side reduction [25,48,49] that is different from the reduction of Fe ions. The cycle performance in Figure 4e shows a capacity increase in the initial few cycles, which is similar to the capacity increase in the first 10 cycles in Figure 4b and is attributed to the decreased polarization of the charge–discharge curve (Figure 4a). The essential reason for the capacity increase may be material activation and could be because the initial material is Li-poor Li_2-*x*_FeSiO_4_, which forms Li_2_FeSiO_4_ during cycling [47], or the increased wettability of the material by the electrolyte [16]. In the following cycles, the capacities at 0.5, 1 and 2 C decayed from the highest capacities of 126.2, 114.7 and 105.4 mAh·g^−1^ to 114.2, 101.6 and 79.3 mAh·g^−1^, with the capacity retention rates of 90.5%, 88.6% and 75.2%, respectively. To understand the capacity decay, differential capacity curves of the 33rd and 160th cycles are compared in Figure 4f. The peak intensity of the 160th cycle curve is significantly reduced, indicating that the Fe^2+^/Fe^3+^ redox reaction rate slows down, which may be related to the changes in the material structure and the chemical environment. The previous in-situ XRD results showed that Li_2_FeSiO_4_ undergoes a two-phase transition from Li_2_FeSiO_4_ to LiFeSiO_4_, and from LiFeSiO_4_ to Li*_y_*FeSiO_4_ (0 ≤ *y* < 1) during the charging process [9,50]. Oxidation of oxygen occurs during the second phase transition [9], resulting in a larger structural change than the first phase transition. During the discharge process, a negligible change was observed on the XRD patterns up to a discharge voltage of 1.5 V [50]. After 10 cycles, the ratio of the monoclinic phase decreased, while the ratio of the orthorhombic phase increased in the two-phase mixed Li_2_FeSiO_4_ [8], indicating that the monoclinic phase may be transformed into the orthorhombic phase during cycling. After charging–discharging, a solid electrolyte interface (SEI) layer composed of an electrolyte redox product was formed on the surface of the Li film in the battery (Figure S3), indicating that the chemical environment in the battery has been changed, thus affecting the cycle performance.

**Figure 5:**
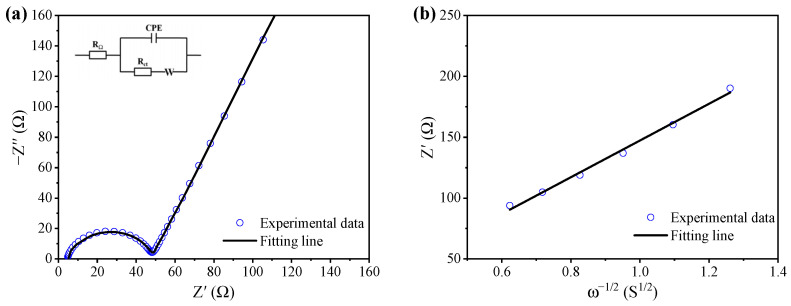
(**a**) Electrochemical impedance spectrum of the LFS600 under open circuit voltage, the inset is the equivalent circuit used to fit the data, and (**b**) linear fitting the data in the low frequency region of (**a**). The frequency values corresponding to the data points in (**b**) are 0.40949, 0.30888, 0.233, 0.17575, 0.13257, 0.1 Hz from left to right.

To analyze the kinetic of the hollow hemispherical Li_2_FeSiO_4_, the electrical conductivity was further identified by EIS characterization, and a Nyquist plot of the LSF600 is shown in Figure 5a. The inset is an equivalent circuit that is used to fit the EIS data. In the circuit, CPE denotes the constant phase element, and is employed to fit the depressed semicircle (an arc). W stands for the Warburg impedance [30], *R*_Ω_ reflects the total resistance including the electrode, separator and electrolyte, and *R*_ct_ is the charge transfer resistance. Fitting the EIS data yields *R*_Ω_ = 4.75 Ω, and *R*_ct_ = 43.1 Ω. Figure 5b is a Z’-ω^−^^1/2^ (ω = 2πf, f is the frequency) plot based on the linear part in Figure 5a, and the slope (σ) obtained by linear fitting can be used to calculate the Li ion diffusion coefficient ($D_{Li^{+}}$) according to the following equations [51,52,53].

(1) $$D_{Li^{+}}=\frac{R^{2}T^{2}}{2A^{2}n^{4}F^{4}C_{Li^{+}}{}^{2}\sigma^{2}}$$

(2) $$Z^{\prime }=R_{\Omega }+R_{ct}+\sigma \omega^{-1/2}$$

where *R* is the gas constant (8.314 J·mol^−1^·K^−1^), *T* is the thermodynamic temperature (corresponding to 298.15 K at room temperature of 25 °C), *A* is the electrode surface area (1.96 cm^2^), and *n* is the number of electron transfers in the redox process (*n* = 1) [30], *F* is Faraday’s constant (96,483 C·mol^−1^), representing the charge carried per mole of electrons, *F* = *e* *N*_A_, *e =* 1.602176 × 10^−19^ C, *N*_A_ = 6.022 × 10^23^, $C_{Li^{+}}$ is the bulk concentration of Li ions in Li_2_FeSiO_4_ (0.04 0.04 mol/cm^3^) [51,52,53]. Substitute the above values into Equation (1) to get the following:

(3) $$D_{Li^{+}}=\frac{5.7680\times 10^{-12}}{\sigma^{2}}$$

The calculated Li ion diffusion coefficient of the LFS600 is 2.53 × 10^−16^ cm^2^·s^−1^, which is close to 2.90 × 10^−16^ cm^2^·s^−1^ for the graphene-modified Li_2_FeSiO_4_ [2].

## 4. Conclusions

In summary, a hollow hemispherical Li_2_FeSiO_4_ was synthesized by a template-free hydrothermal method with the addition of ascorbic acid. In the presence of ascorbic acid, Li_2_FeSiO_4_ would finally grow into hollow hemispheres self-assembled by tiny nanoparticles. At different hydrothermal temperatures, the LFS160, LFS180 and LFS200 samples had different particle sizes, and the higher the temperature, the larger the particles. The carbon-coated LFS600 sample exhibited a high discharge capacity of 192 mAh·g^−1^ at 0.2 C, and the average capacities reached 134.5, 115.5 and 93.4 mAh·g^−1^ at 0.5, 1 and 2 C, respectively. Furthermore, the capacity increased with the cycle number in the first few cycles, and then decayed afterwards. From the perspective of redox analysis, the intuitive reason for the capacity decay is that the redox peak intensity of Fe ions decreased; that is, the redox activity was lowered. Adding ascorbic acid in the hydrothermal process could change the self-assembly behavior of the particles, which provides inspiration for the synthesis of Li_2_FeSiO_4_ nanoparticles with a tuned morphology and an enhanced performance.

## Data Availability

The datasets generated during and/or analyzed during the current study are available from the corresponding author on reasonable request.

## Supplementary Materials

The following supporting information can be downloaded at: https://www.mdpi.com/article/10.3390/ma15103545/s1, Figure S1: Particle size distributions (a) LFS160, (b) LFS180 and (c) LFS200 samples based on 100 particles counted for each sample; Figure S2: Rate performances of LFS600 and nano-LFS600 samples obtained by the hydrothermal reaction with and without the addition of ascorbic acid, respectively; Figure S3: SEM image of the Li film in the charged–discharged LFS600 battery. The substances originated from the redox product at the electrolyte were deposited on the Li film to form an SEI layer; Table S1: Comparison in the maximum capacity of Li_2_FeSiO_4_/C with different spherical morphologies.
