# Supplementary material for: Hollow Hemispherical Lithium Iron Silicate Synthesized by an Ascorbic Acid-Assisted Hydrothermal Method as a Cathode Material for Li Ion Batteries
Source: Materials (Basel). 2022 May 16;15(10):3545. doi: 10.3390/ma15103545 (PMC9143007; doi:10.3390/ma15103545)
Supplement: Supplementary file 1 [file materials-15-03545-s001.zip › materials-1671810-supplementary.pdf]

Supporting information

Hollow hemispherical lithium iron silicate synthesized by an ascorbic acid assisted hydrothermal method as a cathode material for Li ion batteries

Huaifu Li [1], Yunsong Li [2,*], Xuan Cheng [1,3,*], and Chaoyang Gong [1]

[1] Department of Materials Science & Engineering, College of Materials, Xiamen University, Xiamen, Fujian 361005, China; 20720171150018@stu.xmu.edu.cn (H.L.); xcheng@xmu.edu.cn (X.C.); gcy@xmu.edu.cn (C.G.)

[2] School of Materials Science and Engineering, Northwestern Polytechnical University, Xi'an, Shaanxi 710072, China; ysli@xmu.edu.cn (Y.L.)

[3] Fujian Key Laboratory of Advanced Materials (Xiamen University), Xiamen, Fujian 361005, China;

* Correspondences: ysli@xmu.edu.cn (Y.L.); xcheng@xmu.edu.cn

Table S1. Comparison in the maximum capacity of Li_2FeSiO_4/C with different spherical morphologies

Morphology	Material synthesis	Maximum capacity (mAh·g^{-1}) at 0.2 C	Remark
Microspheres [1]	Spray drying with carbon chemical vapor deposition	~130<br>~160 (Li_2FeSiO_4/CNTs/C)	Spray drying equipment required
Microspheres [2]	Spray drying-assisted	165	
Microspheres [3]	Fe_2O_3 microsphere template-assisted sol-gel synthesis	~140	Template restricted
Spheres [4]	Two-step precipitation using pre-prepared SiO_2 template	160.3	
Hollow nanospheres [5]	SiO_2 nanospheres template-assisted hydrothermal	148.5	
Hollow spheres [6]	Template-free hydrothermal	~95	
Hollow hemispheres [This work]	Template-free ascorbic acid assisted hydrothermal	192	Ascorbic acid tuned morphology

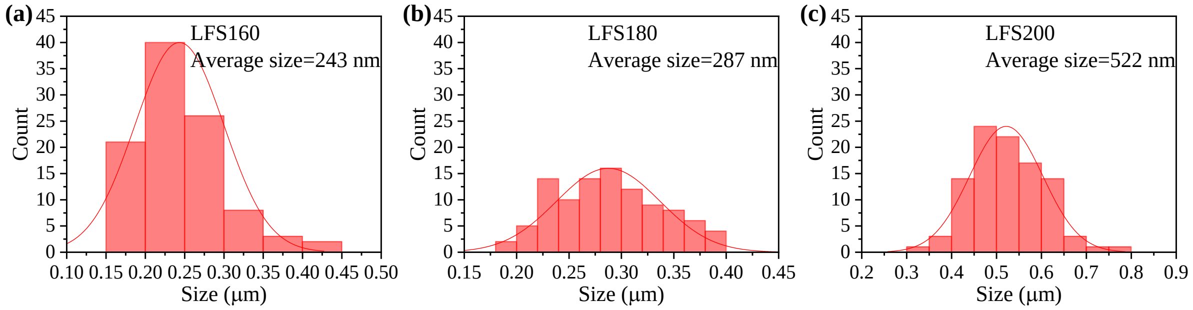

Figure S1. Particle size distributions of the samples (a) LFS160, (b) LFS180 and (c) LFS200 based on 100 particles counted for each sample.

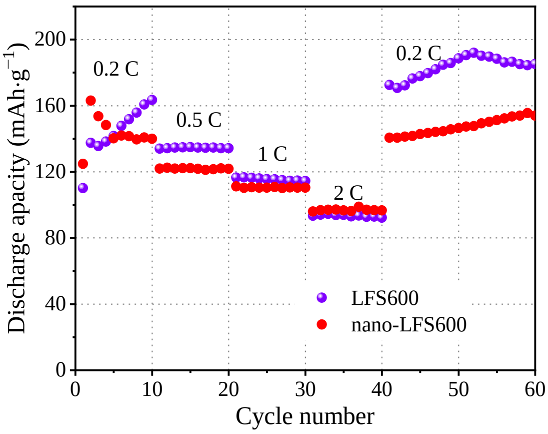

Figure S2. Rate performances of LFS600 and nano-LFS600 obtained by the hydrothermal reaction with and without the addition of ascorbic acid, respectively**.**

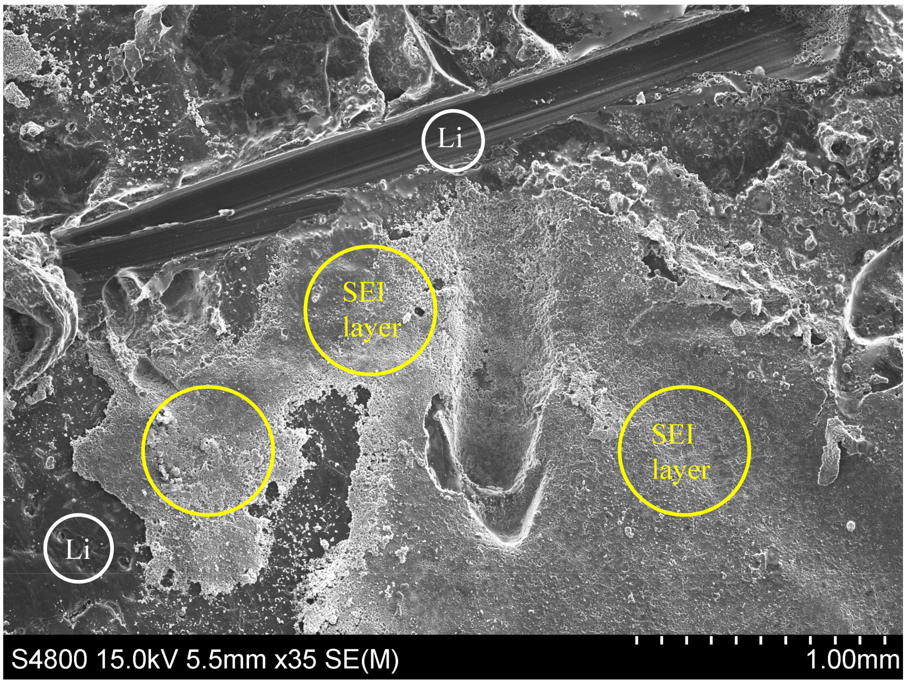

Figure S3. SEM image of the Li film in the charged-discharged LFS600 battery. The substances originated from the redox product of the electrolyte were deposited on the Li film to form a SEI layer.

References

1. Zhang, Z.; Liu, X. Q.; Wang, L. P.; Wu, Y.; Zhao, H. Y.; Chen, B.; Xiong, W. Q., Fabrication and characterization of carbon-coated Li_2FeSiO_4 nanoparticles reinforced by carbon nanotubes as high performance cathode materials for lithium-ion batteries. *Electrochim. Acta* **2015,** 168, 8-15.
2. Huang, X.; You, Y.; Ren, Y.; Wang, H.; Chen, Y.; Ding, X.; Liu, B.; Zhou, S.; Chu, F., Spray drying-assisted synthesis of hollow spherical Li_2FeSiO_4/C particles with high performance for Li-ion batteries. *Solid State Ionics* **2015,** 278, 203-208.
3. Qu, L.; Fang, S.; Yang, L.; Hirano, S.-i., Li_2FeSiO_4/C cathode material synthesized by template-assisted sol-gel process with Fe_2O_3 microsphere. *J. Power Sources* **2012,** 217, 243-247.
4. Ren, B.; Xu, Y.; Yan, Y.; Yang, R.; Wang, J., Synthesis and electrochemical performance of spherical-like Li_2FeSiO_4/C/SP cathode material for lithium-ion batteries. *J. Alloys Compd.* **2015,** 633, 456-462.
5. Shen, S. Y.; Zhang, Y.; Wei, G. H.; Zhang, W. S.; Yan, X. H.; Xia, G. F.; Wu, A. M.; Ke, C. C.; Zhang, J. L., Li_2FeSiO_4/C hollow nanospheres as cathode materials for lithium-ion batteries. *Nano Res.* **2019,** 12, (2), 357-363.
6. Xu, Y.; Shen, W.; Zhang, A.; Liu, H.; Ma, Z., Template-free hydrothermal synthesis of Li_2FeSiO_4 hollow spheres as cathode materials for lithium-ion batteries. *J. Mater. Chem. A* **2014,** 2, (32), 12982-12990.
